# Vagus Nerve Stimulation for Neuromodulation: Evolution from Bench to Bedside

**DOI:** 10.3390/neurolint18050094

**Published:** 2026-05-15

**Authors:** Prasad Vannemreddy, Konstantin V. Slavin

**Affiliations:** 1 Department of Neurosurgery, University of Illinois at Chicago, Chicago, IL 60612, USA; prasad4458@hotmail.com; 2 Section of Neurology, Jesse Brown Veterans Administration Medical Center, Chicago, IL 60612, USA

**Keywords:** vagus nerve, neuromodulation, epilepsy, depression, stroke, spinal cord injury

## Abstract

**Background/Objectives**: Vagus nerve stimulation (VNS) has evolved from a laboratory experiment to a standard of care in several neurological disorders like epilepsy, depression and stroke rehabilitation at present. **Methods**: We reviewed the published literature relevant to its origins in animal models leading to various clinical applications. **Results**: Bailey and Bremer published their observations following VNS in animals while further studies established its utility in some forms of epilepsy. Subsequent observations in epilepsy patients treated with VNS revealed the unequivocal improvement in psychological and behavioral disorders. Consequently, VNS received approval for its application in resistant depression disorders. Multiple studies revealed changes due to neuronal plasticity following VNS that could result in the significant clinical recovery of motor function in chronic ischemic stroke patients. Chronic incomplete cervical spinal cord injury, head injury and peripheral nerve injury deficits are also being studied for recovery patterns. Transcutaneous approaches and closed-loop stimulation are showing encouraging results that may facilitate the extension of the application of neuromodulation using VNS. **Conclusions**: For the recovery of motor function following paralysis in stroke patients or cervical spinal cord injuries, the timing of the stimulation after physical activity during rehabilitation has been identified as a key factor. In addition to the timing of the stimulation, the titration of the parameters is also being studied to obtain optimized recovery in cases of motor, sensory, or sphincter deficits.

## 1. Anatomy of the Vagus Nerve and Neuromodulatory Projections

**Figure 1:**
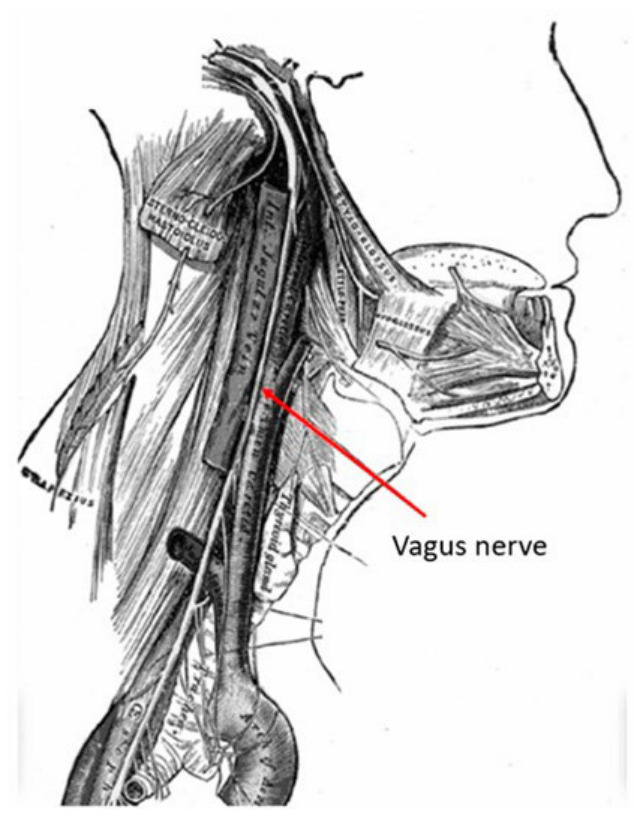
Schematic illustration of the vagus nerve anatomy.

The tenth cranial nerve (the vagus nerve) carries major parasympathetic innervation throughout the body along with motor and sensory fibers. In the neck, it travels within the carotid sheath and is located posterior to the carotid arteries (Figure 1) [1]. The vagus nerve engages several components of the central nervous system by means of the afferents fibers, when stimulated. The first such effect of stimulation was reported by Bailey and Bremmer [2].

Further refined laboratory methods established the connections of the vagus nerve with the Nucleus Tractus Solitarius (NTS), locus coeruleus, raphe nucleus and nucleus basalis, major production sites for neurotransmitters like norepinephrine, acetyl choline (Ach) and serotonin [3,4].

The entire neural axis has connections with these nuclei with highly specific temporal preferences since adrenergic innervations reach prefrontal, precentral and postcentral gyri [5], while serotoninergic connections preferentially innervate the visual cortex and other primary sensory locations [6,7] and Ach fibers innervate limbic, cingulate and paralimbic structures [8].

The spinal cord also receives connections from the locus coeruleus and raphe nuclei providing serotonin and adrenaline [9].

The vagus nerve reaches all these areas of brain and spinal cord via its neurotransmitter projections since the stimulation of this nerve directly influences its nuclei to release norepinephrine, serotonin and Ach in abundance, resulting in the neuromodulation of specific areas [10].

## 2. Material and Methods

The literature on VNS was collected using the key words as above from PubMed, clinicaltrials.gov and Google Scholar. The material included comprised relevant laboratory studies and clinical experience up to January 2026.

## 3. Preliminary Laboratory Evidence

Cortical localization and functional neurosurgery have strong roots in Chicago, where Bailey and his colleagues performed several seminal experiments. With Bremer, he conducted electrical stimulation studies on cats and recorded the activity from the cortex along with the “cardiomoderator reflex” and changes in blood pressure [2]. However, with the primitive equipment, the cortical effect was not distinguishable from a cardiac effect since the vagus nerve also carries fibers to heart. Zanchetti et al. utilized more refined equipment on the same animal model and recorded alterations to the cortical discharges following VNS [11]. Once the electroencephalogram (EEG) was introduced into neurological practice, further recordings were possible, although in a moderate, inconclusive manner [12].

### Central Effects of VNS in Animal Models

Zabara [13] studied experimental seizures in dogs and demonstrated that stimulating the vagus nerve repeatedly aborted the seizure activity, suggesting that small unmyelinated fibers with slow electrical conduction might be the carriers of the effective nerve signals in VNS. However, the selective sectioning of these fibers did not stop VNS from being an effective method to stop seizure activity [14], thus suggesting the additional involvement of other areas like NTS, reticular formation, and dopaminergic pathways [15].

Further studies using immunostaining techniques identified active areas engaged in VNS and seizure abortion: the vagus nuclei, locus coeruleus, NTS, cochlear nuclei, amygdala and other limbic system components apart from habenula and hypothalamus [16]. There is also experimental evidence showing that VNS fails to stop seizure activity in rodents when the noradrenergic locus coeruleus was sectioned, or with the lesioning of serotonin-producing dorsal raphe nuclei [17,18]. Additionally, VNS-induced anti-depression results were also inhibited with lesions of locus coeruleus [19].

During these studies, investigators observed that neurotransmitters were also playing a role in the neural plasticity induced by VNS, especially via norepinephrine, Ach and serotonin [20,21].

It was also emphasized in these studies that stimulation parameters need to be optimized, as there is no monotonic rule for the responses versus a higher frequency or pulse width, while timing the release of neurotransmitters requires careful optimization [10,22,23].

In a laboratory setting, VNS has been tested in relation to many neurological disorders.

## 4. VNS for Epilepsy

### 4.1. Epilepsy Animal Models

The introduction of EEG needs to be acknowledged in relation to the breakthrough achievements of neuromodulation in epilepsy, since it provided direct cortical recordings with and without stimulation [24].

A seminal work by Zabara demonstrated that VNS had a long-lasting effect of seizure control, even after the cessation of stimulation, an observation recorded after repetitive studies at different stimulation parameters [13,25]. Another group tested this experimentation repeatedly in primates, this time in monkeys, with a variety of stimulation combinations [26] and the results were similar. At some point, these laboratories utilized different stimulation ranges in rodents, canines and primates that were equivalent to VNS in humans [14,27].

### 4.2. Surgical Procedure of Implantation of Vagus Nerve Stimulator

**Figure 2:**
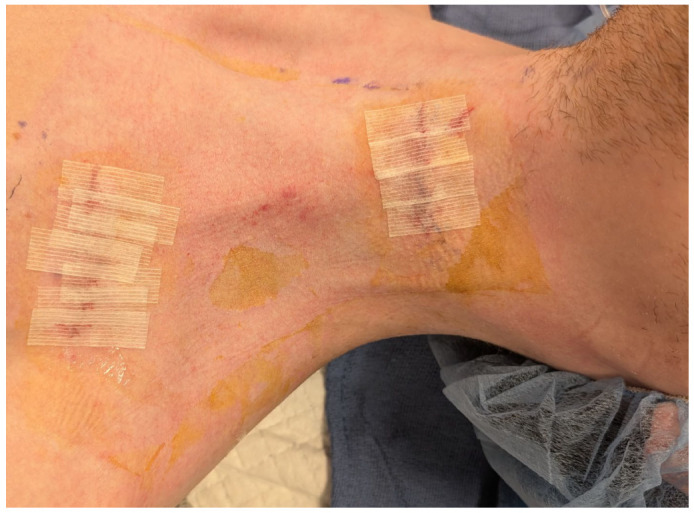
Surgical incisions for the implantation of the VNS device.

The implantation of the vagus nerve stimulator follows an anterior cervical approach (more commonly performed for anterior cervical discectomy and fusion). The incision is given on the left side to reach the carotid sheath, where the vagus nerve is identified in its posterior layer next to the sympathetic chain. The nerve is longitudinally dissected to create enough space to entangle the spring electrode wrapped around it. The electrode is next connected to the pulse generator. A separate infraclavicular incision is performed to create a pouch to accommodate the pulse generator. These two incisions are connected via a subcutaneous tunnel (Figure 2). The vagus nerve stimulator, the connecting leads and the pulse generator are verified to be actively communicating followed by closure of the incisions.

The procedure is performed under general anesthesia and has a very low incidence of intraoperative morbidity or mortality. In their long-term follow-up with nearly 500 patients, the Ben-Menachem group reported postoperative hematoma and infection as the common complications. The overall complication rate was below 10% and hardware-related adverse events occurred in 3.7% of patients [27].

### 4.3. VNS for Epilepsy in Clinical Studies

Several experimental studies revealed the effectiveness of VNS in controlling seizures in different animals. However, in human epilepsy situations, Penry and Dean published the first preliminary results in 1990 [28], after testing a variety of stimulation parameters. A different combination of the VNS neuromodulation strategy was applied by Uthman et al., providing on and off stimulation methods [29]. This was followed by individualized stimulation readings by Wilder et al. in more patients, which demonstrated the safety and feasibility of VNS with promising results [30]. These studies provided important information regarding the long-lasting effects of VNS even without stimulation. Thus, this paved the way for further scientific research with RCT by Ben-Menachem et al. [31,32]. This study over a period of 14 weeks established VNS as an effective treatment for seizures and patients were able to tolerate the stimulation.

Further clinical studies have established the efficacy of VNS in controlling the frequency of seizures [33,34,35]. George et al. [33] formed the VNS study group for a randomized controlled study of chronic VNS especially for refractory seizure management, while Handforth et al. [34] studied partial onset seizures. A meta-analysis of stimulation parameters versus acute response in pediatric cases of epilepsy was published by Ghani et al. [35]. High-frequency stimulation was more effective compared to low-frequency stimulation in adults, while children did not exhibit any significant difference in outcome. Nevertheless, high-frequency stimulation had higher incidence of hoarseness and dyspnea. The titration of ON and OFF durations also yielded optimum results. In their retrospective analysis, DeGiorgio et al. could not establish a significant association between adjusting the stimulation parameters and treatment outcome in two studies [36,37].

## 5. VNS for Refractory Depression

Elger et al. noted that VNS treatment for epilepsy affected mood also apart from seizure activity [38]. These authors went on with analyzing their observations on a larger number of patients to conclude that patients improved in their mood alterations irrespective of their response to VNS for epilepsy. Similarly other authors also established the efficacy of VNS in refractory depression [39], along with fMRI documentation [40,41,42,43].

George et al. compared the effects of VNS implant therapy with the standard of care in non-invasive conservative treatment. They received VNS combined with conservative treatment and the latter alone (without VNS) to demonstrate that the first group had a significant improvement (27% vs. 13%), paving way to an open label trial followed by the FDA approval of VNS for refractory depression in 2007 [44]. The supporting evidence for VNS indications in refractory depression has been steadily increasing and a larger number of patients are receiving or being recruited in RCT (RECOVER trial) with approvals for therapy.

Additionally, investigators have further studied the role of VNS in neural plasticity to improve other cognitive functions apart from changing the course of epilepsy.

## 6. The Substrate for Neural Plasticity with VNS in Motor Function Recovery

The role of neurotransmitters in neuroexcitation and neural plasticity has been established in research. Norepinephrine, serotonin and acetyl choline play a key role in modulating this activity and Engineer et al. studied VNS induced changes in rodent models [45,46,47,48,49,50]. Experimental models also analyzed the alterations in stimulation parameters vs the neuromodulation benefits of VNS [50]. The exhaustion of neurotransmitters aborted the VNS-induced neuronal plasticity and its benefits [51,52,53]. This neurotransmitter network is modulated by VNS in order to improve motor recovery in stroke, especially when the upper extremity motor function is the target [20,47].

## 7. VNS-Paired Plasticity in Preclinical Studies

Norepinephrine networking played a role when paired VNS was used for rehabilitation in rodent brain injury models [53]. Cortical engagement was demonstrated when VNS was applied for 5 days, demonstrating high degree of correlation with improved motor function even in healthy models [54]. More importantly, primed animals (those receiving paired VNS before ischemic events) demonstrated faster improvement in their motor weakness. Porter et al. [54], Engineer et al. [47] and Meyers et al. [55] also observed that these outcomes following paired VNS continued for several months post-treatment. Even in cervical spine injury rodent models, Darrow et al. [56] reported a significant improvement in somatosensory deficits.

## 8. Clinical Application of Paired VNS for Stroke Rehabilitation in Adult Human Beings

Cerebrovascular accidents and the neurological deficits that follow brain stroke have been quite challenging to manage in clinical settings. Thus, VNS and its neural plasticity effects received attention very promptly, leading to clinical trials of paired VNS in ischemic stroke patients compared to routine rehabilitation methods. Patients who received paired VNS had shown significant recovery in upper extremity motor function in 6 weeks [57]. There was also improvement in terms of tactile sensations in chronic stroke patients [58].

This was followed by a sham stimulation study wherein ischemic stroke patients who received paired VNS rehabilitation demonstrated considerable improvement in their Fugl–Meyer assessment upper extremity (FMA-UE) scores and Wolf motor function test [59].

In VNS-REHAB study with large group of ischemic stroke patients, Dawson et al. confirmed the positive influence of paired VNS rehabilitation in recovering the motor function [60].

## 9. Benefits of VNS in Therapy of Non-Motor or Non-Stroke Neurological Deficits

VNS neuromodulation is being studied for its effects in experimental models of cervical spinal cord incomplete injuries [61] and also in nerve injury models [53] with encouraging results. There are other laboratory observations proposing the utility of VNS even in sphincter malfunction, speech and swallowing deficits [62]. We reviewed the utility of VNS in stroke rehabilitation recently [63].

## 10. Role of Non-Invasive VNS in Neural Plasticity

VNS has been rapidly evolving from bench to bedside as a standard of care in neuromodulation, and simultaneous work also started exploring the nonsurgical utility of vagus nerve stimulation. One such utility was related to the stimulation of the nerve in its carotid sheath (tcVNS) and a second utility looks at engaging the auricular branch at the ear (taVNS). Attempts to compare these non-invasive methods to surgical VNS have not disclosed comparable results; most probably due to dissimilar pathways involved in these techniques [64,65]. In addition to the failure to access the original vagus nerve pathways, it is also possible, as demonstrated in animal studies, that the cutaneous stimulation requires much higher stimulation intensity, very likely intolerable, to yield clinically comparable results to the implanted VNS [66].

Non-invasive VNS also faces the challenge of differences in the anatomy of the nerve in patients, impacting the delivery of the desired amount of stimulation to obtain perceivable clinical results [67,68].

There are other confounding variables like the impedance of the skin and underlying tissues that vary with every patient, and neck movements from daily activities that keep shifting the location of the equipment. Chang et al. [69] and Li et al. [70] reported equivocal results from their clinical pilot studies in this field.

However, non-invasive VNS offers better patient acceptance and preference since surgical discomfort and complications do not exist.

## 11. Discussion

The vagus nerve, known as the vagabond cranial nerve, is the longest cranial nerve that supplies variable visceral and somatic structures in the body. It is located in the posterior part of the carotid sheath in the neck on its way to the thorax and abdomen (Figure 1).

The exploration of the electrical activity of this nerve started in Chicago, when Percival Bailey performed stimulation studies in his laboratory while experimenting on cerebral localization in 1938. Along with Bremer, he published his observations on the cortical projections of the vagus nerve to sensory areas via the thalamus, thus opening the possibilities of VNS for neuronal plasticity [2].

The electrophysiological studies conducted by Bailey paved the way to the surgical treatment of epilepsy when he performed the first ever anterior temporal lobectomy for refractory epilepsy utilizing intraoperative electroencephalography in 1946 [71]. Today VNS has become a standard of care for the surgical treatment of chronic epilepsy: both entities (VNS and epilepsy surgery) were introduced by neurological research from Chicago.

### 11.1. Epilepsy

Seizure disorders are one of the most common neurological ailments affecting over 50 million people in the world with an addition of 5 million newly diagnosed cases annually. Nearly 20–40% of these new cases turn from refractory to medical management [72].

Thus, the requirement for alternative modalities of treatment exists for epilepsy disorder. All the trials conducted to establish the safety and efficacy of VNS have provided supporting evidence, and there is no interference of neuromodulation with commonly used antiepileptic medications. Hence, VNS received approval from the FDA for the management of refractory partial seizures in 1997 [73]. The successful reduction in seizure activity following VNS expanded its indications to younger adults and children presenting with other kinds of epilepsy, including Lennox–Gastaut syndrome [74].

Most of the clinical experience with VNS comes from its applications for epilepsy, although additional patients with depression and stroke have received this treatment. Complications from the implantation of VNS have been reported and the major ones, including hardware complications, occurred in up to 8.6% of 497 patients from Ben-Menachem group. Of these, 3.7% were hardware-related complications. The most common were postoperative hematoma and infection while vocal cord paralysis, facial weakness, pain and lead- or battery-related complications also occurred [28]. Nevertheless, the results have overwhelmingly convinced patients and families to accept VNS as an option for refractory epilepsy.

### 11.2. VNS for Major Refractory Depression

Disabling major depression has become a common diagnosis, and in recent years its incidence has increased by a 7.1% prevalence per year [75].

Nearly one third of these patients do not appear to respond to the standard of care management that includes psychotherapy with medications [76].

The socio-economic burden of this disorder is so expensive that the demand for new methods of treatment is very high. Evidence from experimental models and neuroimaging studies has demonstrated the potential beneficial effects of VNS in terms of the modulation of cortical areas, its effects on monoamine pathways and, more importantly, the relief reported in epileptic patients with depression following VNS [77]. In early trials, positive outcomes were reported when VNS was used for depression [78,79].

Rush et al. conducted a multisite enrolment of 40 patients with refractory depression. VNS was initiated after 2 weeks and modulated over the next 2 weeks for tolerability, followed by 8 weeks of neuromodulation. At the end of 3 months nearly half of them experienced a reduction in symptoms. An additional 30 patients were included after 3 months [78,79].

These authors reported a lasting relief from depression even at the end of 1 year with significant improvements in remission. Others noticed that VNS induces a sustainable, gradual response in Montgomery–Åsberg Depression Rating Scale, or in MADRS compared to the controls [80].

Nevertheless, the equivocal results from some studies limit the indications for VNS-treated depression at present and further observations might increase the utility of the modality [80,81,82]. To answer some of these concerns, the FDA recently approved the re-evaluation of dedicated VNS devices through large-scale clinical trials.

### 11.3. VNS in the Management of Stroke

Paired VNS neuromodulation evolved independently of the epilepsy treatment as a closed-loop method to achieve the coordination of the stimulation with motor activity during rehabilitation [83]. The neural plasticity of this paired VNS engages noradrenergic, serotonergic and cholinergic networks to enhance the synaptic activity induced by physical training [65].

In animal models, there was a reproducible motor improvement of over 50% when paired VNS was compared with sham stimulation for upper limb function [84].

Three human stroke studies followed these pre-clinical observations to prove the safety and efficacy of this in ischemic stroke [57,59,60].

The third study by Dawson et al. [57] was a sham-controlled study with randomization that included 100 patients receiving 18 rehabilitation sittings of about 27 h. This study confirmed the utility of paired VNS by providing unequivocal evidence of the significant improvement in motor function measured by the Fugl–Meyer assessment (FMA), which was meaningful for the patients in terms of their improved activity as seen in the Wolf motor function test at the 3-month follow-up. The changes were prominently noticed in relation to distal dysfunction [85].

### 11.4. The Essence of Timing in Paired VNS

There is neuromodulatory reinforcement operating behind the coordination of VNS with active rehabilitation.

The engagement of learning and memory necessitates the timing of reinforcement in terms of paired stimulation; this is so significant that a delay in this catalyst action reduces the impact of the overall therapy [86].

The synaptic activity is memorized at the cellular level for reinforcement depending upon the timing of the stimulation: earlier neuronal stimulatory activity yields an efficient output for stronger plasticity signals, unlike a delayed stimulation [87].

In the laboratory, cortical slices demonstrated that within a few seconds following the activation of synapses inducing release of norepinephrine or serotonin, the effective longevity of the potentiation results occurred [88]. In paired VNS, specific timing was recorded within 2 s following the rehabilitation stimulus to obtain improved plasticity and neurological recovery. A delivery delay for tens of seconds fails to elicit these beneficial outcomes [51,89]. Similarly, the delivery of the VNS has to follow the rehabilitation activity since an early input had no effect [90].

Apart from the neurotransmitters, VNS also provides anti-inflammatory effects, reduces programmed cell death (apoptosis) and also modulates blood–brain barrier permeability [91]. These neuroprotective mechanisms might be useful in acute stroke management.

Animal studies, however, have ruled out any contribution from dopaminergic pathways that drive the reward mechanisms [92,93].

### 11.5. Neural Network Mechanisms of Paired VNS for the Treatment of Motor Deficits

In terms of paralyzed upper extremity recovery, VNS engages the corticospinal tract on the same side and the opposite side cortico-reticulospinal tract [94,95]. In rodent models, paired VNS demonstrated much better recovery in the form of increased connectivity in both the side sensory and motor cortex, compared to sham stimulation controls [94]. In other models, there was also increased neuronal plasticity observed following VNS [55,95].

### 11.6. Non-Invasive Paired VNS

Highlighting the complications that follow surgical procedures, and encouraged by the non-invasive transcutaneous stimulation technology, non-invasive paired VNS received attention. The targets have included the auricular branch of the vagus nerve at the external ear and the cervical trunk of the vagus nerve in carotid triangle.

Two closed-loop transcutaneous auricular nerve stimulation systems have been developed: Respiratory-gated Auricular Vagal Afferent Nerve Stimulation (RAVANS) [95] and Motor-Activated Auricular Vagus Nerve Stimulation (MAAVNS) [96].

RAVANS delivers stimulation for 500 milliseconds in response to exhalation and waits until the next cycle of respiration, as inhalation leads to inhibition of the vagus nerve temporarily. As a closed-loop system tested in patients with chronic pain and migraine, this was promising in its results [95]. These patients were compared to healthy controls by using fMRI of the brainstem to evaluate the activity of NTS. There were several confounding factors in this study, and the sample size was a major limiting factor.

MAAVNS is more like the paired VNS, engaging motor activity coordination, and recent reports by Badran et al. promise an effective non-invasive modality [96,97].

Redgrave et al. and Baig et al. demonstrated effective taVNS in the rehabilitation of stroke patients with an upper extremity weakness [98,99]. Capone et al. utilized robotic rehabilitation using transcutaneous VNS [100]. At present, a randomized clinical trial is on its way using the MAAVNS technology for upper extremity rehabilitation in adults [101].

With tcVNS, Badran et al. conducted the first pilot study in 16 patients examining rehabilitation following stroke, and reported results comparable to implanted VNS [102]. In their report, the authors also concluded that stimulation timing is important; stimulation with pairing was superior to unpairing (similarly to the implanted VNS protocol).

### 11.7. VNS and Spinal Cord Injury Management

For incomplete cervical spinal cord injury patients with an upper extremity weakness, a clinical trial to utilize closed-loop VNS (CLV) was registered recently, based on preclinical evidence [103]. This is a prospective, double-blinded, sham-controlled and randomized study. It uses gamified physical therapy with force and motion sensors for sham and active stimulation. In its initial report after 12 weeks of treatment with the miniature CLV paired stimulation, 19 spinal cord injury patients with incomplete paralysis exhibited significant improvements in hand function that improved their quality of life [61]. Another study registered with clinical trials from Houston; Texas has not reported any activity after the protocol publication [104]. This trial had a proposal for 6 weeks of in-clinic VNS paired with rehabilitation [105].

Complications following VNS implantation have been both rare and tolerable. The Ben-Menachem group [27] reported their experience with epilepsy management. While the overall complication rate was below 10%, which included wound hematoma-, infection- and implant-related adverse events, hardware-related issues like lead migration occurred in 3.7%. A review of VNS-associated events included voice changes, a cough and sometimes breathing difficulty. The most common was voice hoarseness (45.5%), while vocal cord paralysis occurred in 1.4% to 5.6% [106].

## 12. Conclusions

VNS is a standard of care for the surgical treatment of refractory epilepsy today. Both electrical stimulation of the vagus nerve and surgical treatment for psychomotor epilepsy have roots in Chicago and the neuropsychiatry team of Percival Bailey.

Tested in relation to epilepsy management, implantable VNS is receiving attention for its promising neuronal plasticity effects made possible by its extensive afferent network stimulating norepinephrine, serotonin and cholinergic pathways.

VNS paired with rehabilitation to improve functionality of stroke patients has demonstrated encouraging results in its preclinical and clinical trials boosted by vast experimental data. This promising modality has many parameters to be explored to obtain reproducible clinical results and wider indications in varied neurological disorders that have dismal prognoses at present. Resistant depression, stroke, and neurotrauma are some of the many potential examples.

VNS includes rehabilitation training and electrical stimulation, which require the appropriate combination for maximum utility. A variety of possibilities exist [107,108]. In the past, we have investigated and reported on the development of adaptive deep brain stimulation (DBS) in tremor patients using surface electromyography [109]. However, similar applications for VNS have not yet been reported.

### Future Directions

Neuronal plasticity studies recommend timing as the essence of this paired VNS therapy, and neuromodulation needs reinforcement in order to engage the appropriate neurotransmitter pathways that contribute to recovery. In this context, further studies are required to explore the possibility for the identification of prognostic indicators and the specifics of stimulation as well as rehabilitation techniques.

In the neuroplasticity arena, future work can investigate the relationships between current intensity, pulse width, and frequency for optimum outcomes. However, as emphasized above, no single combination of parameters exists in VNS for optimum outcomes in any of the above-discussed disease entities. Thus, this status opens up possibilities for a wider spectrum of stimulation variables.

Everybody is different.

## Data Availability

No new data were created.

## Abbreviations

The following abbreviations are used in this manuscript:

Ach	Acetylcholine
EEG	Electroencephalography
FDA	Food and Drug Administration
FMA-UE	Fugl–Meyer Assessment Upper Extremity
MAAVNS	Motor-Activated Auricular Vagus Nerve Stimulation
NTS	Nucleus Tractus Solitarius
RAVANS	Respiratory-gated Auricular Vagal Afferent Nerve Stimulation
RCT	Randomized Controlled Trial
taVNS	Transcutaneous Auricular Vagal Nerve Stimulation
tcVNS	Transcutaneous Cervical Vagal Nerve Stimulation
VNS	Vagus Nerve Stimulation
